# Lipoma of the gastrointestinal tract: a tertiary care centre experience

**DOI:** 10.1308/rcsann.2023.0063

**Published:** 2023-10-12

**Authors:** S Kumar, AG Harisankar, R Singh, A Kumar, B Kumar, M Mandal

**Affiliations:** ^1^ Indira Gandhi Institute of Medical Sciences, India; ^2^ BIG Apollo Spectra Hospitals, India; ^3^ Sri Krishna Medical College and Hospital, India

**Keywords:** Benign tumours, Haemorrhage, Gastrointestinal, Intussusception, Lipoma

## Abstract

**Introduction:**

Gastrointestinal (GI) lipomas are rare; however, they are frequent enough to be considered in the differential diagnosis of gut tumours. Here, we present our experience with GI lipomas managed at our institute over the last three years.

**Methods:**

This is a retrospective cohort study of patients with GI lipomas managed between January, 2020 and April, 2023 at a tertiary care centre. Clinical presentation, location, and details of surgical procedure were analysed.

**Results:**

Ten patients were included, six of whom had lipoma in the colon, one in the stomach, and one each in the duodenum, jejunum, and ileum. The mean age at the time of presentation was 48.8 years (range, 19–77 years), and strong male preponderance (4:1) was noted. Preoperative diagnosis of lipoma on cross-sectional imaging was possible in all patients. All patients were symptomatic and were managed surgically.

**Conclusions:**

While GI lipomas are generally considered to be indolent and benign tumours, they can potentially lead to severe complications. The utilisation of computed tomography and magnetic resonance imaging has brought about a significant transformation in diagnosing this condition, enabling preoperative identification in most cases. The surgery offers a definitive treatment with minimal risk of postoperative complications.

## Introduction

Although considered rare, lipomas are the second most common nonepithelial benign neoplasm of the alimentary tract after leiomyomas.^1^ Most gastrointestinal (GI) lipomas are asymptomatic and are detected incidentally. As the lipoma grows in size, it produces symptoms such as pain, haemorrhage and obstruction. These lesions are seen most commonly in the colon, followed by small intestine, stomach and oesophagus.^2^ The definite preoperative diagnosis of lipoma was not possible before advent of cross-sectional imaging, and hence it was difficult to differentiate lipomas from malignant neoplasms of the gut.^3^ Currently, computed tomography (CT) and magnetic resonance imaging (MRI) allow positive diagnosis by showing the pathognomonic fat density of these tumours.^4^ Surgical resection is usually the most effective option for symptomatic GI lipomas.

In this article, we present our experience with GI lipomas managed at our institute over last three years along with a review of the literature on presentation and management of these rare tumours.

## Materials

After approval by the Institutional Ethics Committee, medical records of patients with GI lipoma managed between January 1, 2020 and May 31, 2023 were reviewed retrospectively. Details of patient characteristics, clinical presentation, operative details and outcomes were retrieved from our prospectively maintained electronic database.

Patients with characteristic fat-attenuating lesion (ranging from −80 to −120 Hounsfield units (HU) on CT scan) with histopathological features of lipoma were included in this study. Patients with mixed histology, such as angiolipomas and fibrolipomas, were excluded.

All patients were managed by surgical excision of lipoma, either by open or laparoscopic approach. A 30-day follow-up record of all patients was maintained for any postoperative complication.

## Results

**Figure 1:**
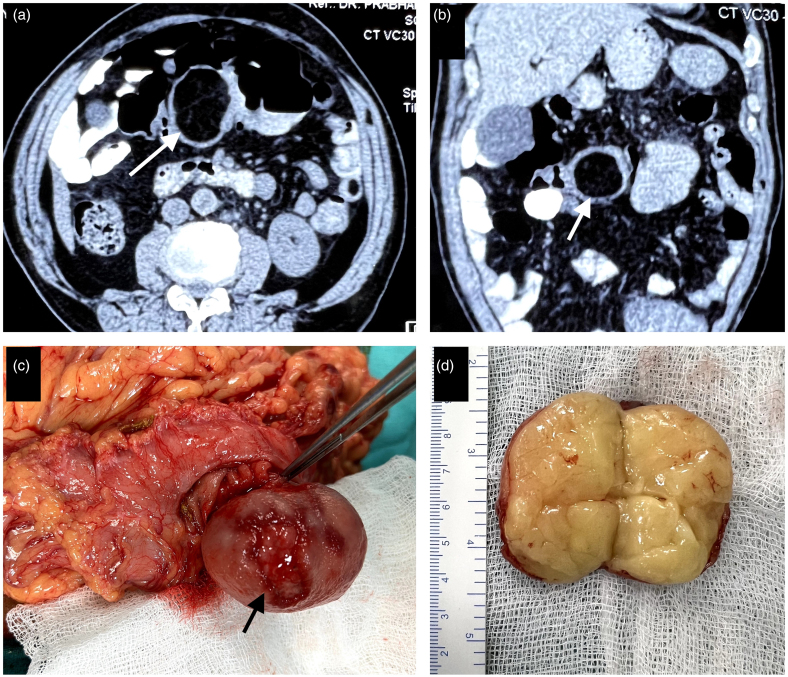
(a and b) Abdominal CT scan images showing lipoma in transverse colon with characteristic fat equivalent lesion density (white arrow). (c) Intraoperative image showing a submucosal lesion with ulcerated mucosa (black arrow). (d) Cut-section examination showing a fat-containing capsulated tumour. CT = computed tomography

Ten patients with GI lipoma were managed between January, 2020 and April, 2023 (Table 1). Eight patients were men and two were women. The mean age at the time of presentation was 48.8 years (range, 19–77 years). Six patients in our series had colonic lipoma (Figure 1) whereas gastric, duodenal, jejunal and ileal lipomas were encountered in one patient each. In all of our patients, a preoperative diagnosis of lipoma could be established based on the characteristic fat attenuation observed on CT scan. The radiological diagnosis corroborated with histopathology as well. The mean maximum axial dimension of lipoma was 4.35cm (range, 3–7cm).

**Table 1:** Table showing patient characteristics: patient demographics, tumour location, size and clinical presentation

Patient No.	Age (years)/Sex	Location	Size of lesion (cm)	Symptoms	Duration of symptoms
1	77/M	Transverse colon	4×4	Abdominal pain Melena	3 months
2	52/F	Hepatic flexure colon	4×4	Abdominal pain	15 days
3	40/M	Descending colon	5×5	Abdominal pain Constipation	3 months
4	52/F	Caecum	5×5	Abdominal pain	2 years
5	72/M	Transverse colon	7×4	Abdominal pain Constipation	1 year
6	40/M	Transverse colon	4×4	Abdominal pain Constipation	6 months
7	54/M	Gastric antrum	5×4	Massive haematemesis	1 day
8	50/M	3^rd^ part of duodenum	3.5×2.5	Abdominal pain Vomiting Melena	2 months
9	19/M	Mid jejunum	3×2	Abdominal pain, Melena	4 months
10	37/M	Mild ileum	3×3	Abdominal pain Massive lower GI bleed	20 days

F = female; GI = gastrointestinal; M = male

**Table 2:** Imaging findings and treatment details of patients with digestive tract lipoma

Patient No.	CECT findings	Procedure	Approach
1	Lipoma in transverse colon	Excision of lipoma	Laparoscopic
2	Colonic lipoma with ileo-colo-colic intussusception	Right hemicolectomy	Laparoscopic
3	Descending colon lipoma with colo-colic intussusception	Left segmental colectomy	Laparoscopic
4	Hepatic flexure lipoma with colo-colic intussusception	Right hemicolectomy	Laparoscopic
5	Transverse colonic lipoma with colo-colic intussusception	Right hemicolectomy	Open
6	Descending colon lipoma with colo-colic intussusception	Segmental transverse colectomy	Open
7	Gastric antral lipoma	Wedge resection	Laparoscopic
8	Duodeno-jejunal flexure lipoma	Excision of lipoma	Open
9	Jejunal lipoma with long segment intussusception	Segmental jejunal resection	Laparoscopic
10	Ileal lipoma with ileo-ileal intussusception	Segmental ileal resection	Laparoscopic

CECT = contrast enhanced computed tomography

All patients in our series were symptomatic for their disease. Abdominal pain was the most common presenting symptom, followed by GI bleed and obstruction. GI bleed was slow and chronic in three patients whereas in two patients it was massive, necessitating emergency surgery. In our series, lipoma was associated with intussusception in seven out of ten patients.

**Figure 2:**
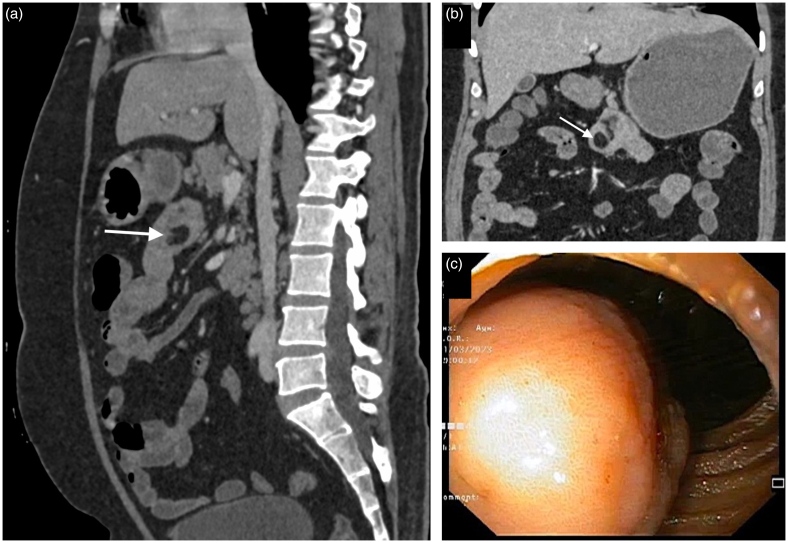
(a and b) Sagittal and coronal sections of abdominal CT scan showing fat-attenuating lesion in duodenum. (c) Endoscopic view showing a smooth submucosal lesion obliterating the duodenal lumen. CT = computed tomography

Four of our patients presented with acute symptoms, two with massive GI bleed and another two with intestinal obstruction. Emergency surgery was performed in these patients. Patients with gastric and midileal lipoma with massive bleed were managed laparoscopically. Patients with colonic lipoma with intussusception and features of acute obstruction needed exploratory laparotomy. One patient with distal duodenal lipoma (Figure 2) also required open surgery due to the challenge of locating and excising the lesion laparoscopically.

**Figure 3:**
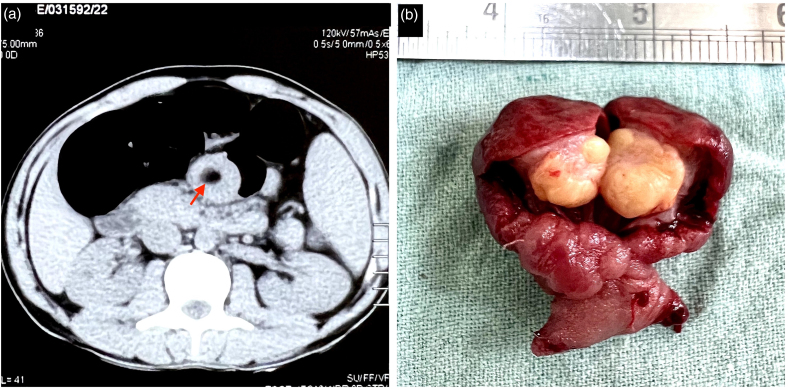
(a) Axial section of abdominal CT scan showing an adipose laden lesion (arrow) in the jejunum. (b) Gross examination of the resected jejunal segment showing a lipomatous lesion. CT = computed tomography

**Figure 4:**
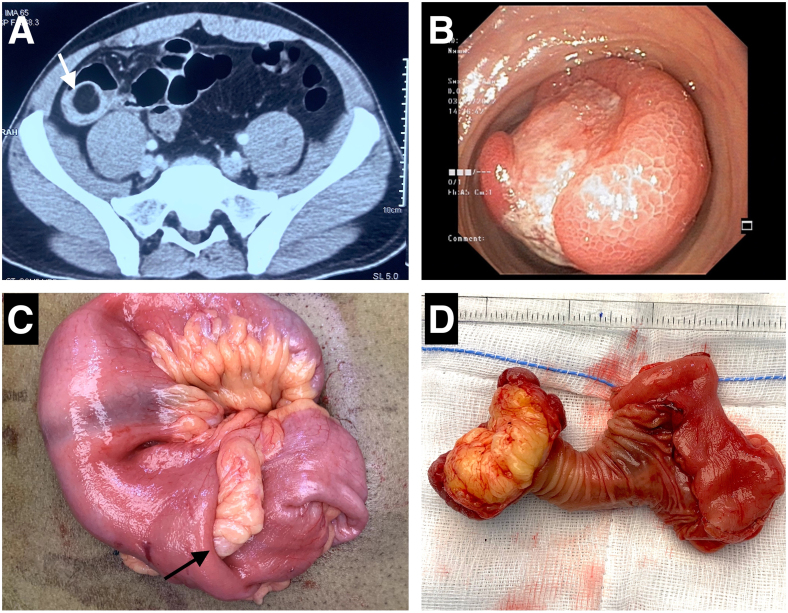
(a) CT scan of abdomen showing an ileal lipoma with ileo-ileal intussusception (arrow). (b) Enteroscopy showing a large subserosal lesion with ulcerated mucosa. (c) Intraoperative finding suggested an ileo-ileal intussusception (arrow) with lipoma as the lead point. (d) Gross examination revealed lipomatous lesion with elongated stalk arising from ileum. CT = computed tomography

The most common procedure performed was resection of lipoma with a segment of bowel. Five out of six patients with colonic lipoma underwent colonic resection. Jejunal and ileal lipomas were also managed by segmental bowel resection with primary side-to-side stapled anastomosis (Figures 3 and 4). In one patient with lesion in transverse colon, lipoma was excised locally without bowel resection. Similarly, patients with duodenal and gastric lipoma were managed by local excision to reduce postoperative morbidity (Table 2).

Postoperative recovery was uneventful in all patients. No major morbidity was recorded in a follow-up of 30 days. Two patients with exploratory laparotomy had surgical site infection that was managed conservatively.

## Discussion

GI lipomas are slow-growing, benign neoplasms composed mainly of mature adipocytes. These are uncommon tumours and constitute about 3% of all GI neoplasms. According to the literature,^5^ lipomas occur predominantly in the colon (60–75%) and the small intestine (30%). Among colonic lipomas, the ascending colon is the most common location (45%), followed by the sigmoid colon (30%), descending colon (15%), and transverse colon (9%).^6^ In our series also, six out of ten patients had colonic lipoma whereas three patients had lipoma in small intestine. We also observed a different distribution of lipoma in colon. Specifically, three cases were located in the transverse colon, two in the right colon and one in the descending colon.

Most lipomas are asymptomatic and diagnosed incidentally during the endoscopic or radiological examination.^2,3^ However, all patients in our series were having symptoms. This is probably due to referral bias, as symptomatic patients are more likely to be referred for surgery. Pathologically, lipomas can be of submucosal, subserosal or intramuscular variant. Approximately 90% of the reported GI lipomas are the submucosal type that present as an intraluminal polypoidal lesion.^7^ All patients in our series had the submucosal type of lipoma.

The majority (50%) of reported small intestinal lipomas occur in the ileum, whereas duodenal and jejunal locations are infrequent.^8^ Our study revealed a homogeneous distribution of lipomas in the small bowel, which may be attributed to the small size of our study sample. The reported peak age of presentation of GI lipomas is in the sixth–seventh decade of life with a female sex preponderance.^1,6,7^ In our series, the mean age of patients was 48.9 years with strong male preponderance (80%). Only two patients were younger than 40 years. Based on these findings, it can be concluded that lipomas typically manifest in individuals after the age of 50 years, during the later stages of life.

GI lipomas usually present with abdominal pain, haemorrhage or features of bowel obstruction.^6,7,9^ Based on previous research, approximately 16–19% of surgeries for GI lipomas are attributed to intussusception, while the remaining cases are linked to intestinal obstruction or haemorrhage.^10^ A total of 90% of our patients had abdominal pain whereas GI bleed was present in 50% of cases; 70% (seven out of ten) patients had associated intussusception on CT scan, with five having obstructive symptoms. In adults, lipomas causing acute intestinal obstruction can manifest in approximately 20% of cases.^11^ Four patients in our series presented with acute symptoms, two with massive GI bleed and another two with acute intestinal obstruction. The latter were the patients with colonic lipoma who presented with intussusception and exhibited obstructive symptoms. Emergency surgery was performed in all these patients.

Before the introduction of cross-sectional imaging, GI lipomas posed a diagnostic challenge as distinguishing them from malignant neoplasms was difficult. Consequently, many patients were presumed to have malignancy and were offered radical surgery. The differential diagnosis of GI lipoma includes various submucosal lipomatous lesions, such as gastrointestinal stromal tumours (GIST), neuroendocrine tumours, and leiomyoma. It is important to consider that malignant tumours, such as leiomyosarcoma, epithelioid endometrial stromal sarcoma and epithelioid angiosarcoma, can also resemble GI lipomas.^12^ Nowadays, diagnostic ability has improved significantly with the widespread use of CT and MRI scans. Advanced diagnostic tools, such as capsule endoscopy, endoscopic ultrasound and double balloon endoscopy, have further enhanced diagnostic accuracy.^1,9^ Accurate preoperative diagnosis of lipoma was made on the basis of endoscopic and imaging findings in all our patients.

Surgery remains the mainstay of treatment for patients with symptomatic GI lipomas.^6,7^ Recently, endoscopic treatment of lipomas has been shown to be a feasible and safe option for select groups of patients. In a comprehensive review focusing on giant gastric lipomas measuring over 4cm, attempts were made to perform endoscopic resection in 2 out of 32 patients, resulting in a reported success rate of 50%.^13^ Another systematic review on the endoscopic treatment of large symptomatic colon lipomas revealed that no adverse events were detected in patients who underwent endoscopic treatment using unroofing and dissection-based resection. However, among patients who underwent endoscopic mucosal resection and loop-assisted techniques, adverse events were observed in approximately 13% of cases.^14^ Similarly, in a systematic review of small bowel lipomas, out of 147 patients, 10 were managed endoscopically, with a reported success rate of 90%.^9^ In the present study, all of our patients received primary surgical management, and endoscopic resection was not attempted in any of the cases.

Currently, laparoscopy has emerged as the preferred surgical approach for the management of GI lipomas. In our series, out of ten patients, seven were successfully treated laparoscopically. However, cases with acute obstructive symptoms or duodenal involvement necessitated open surgery. The majority of patients in our series had large lipomas, often associated with intussusception. As a result, segmental bowel resection was performed as the preferred management strategy in our patients.

## Conclusion

GI lipomas are generally rare and slow-growing tumours but, in rare instances, they can lead to life-threatening complications. Emergent situations may arise with acute bowel obstruction or massive gastrointestinal bleeding. The advent of CT and MRI has revolutionised the diagnosis of this condition, enabling more accurate and timely identification. As endoscopic and laparoscopic techniques continue to advance and become more accessible, the need for open surgery has been on the decline. These minimally invasive approaches offer effective alternatives for managing GI lipomas.
